# Supplementary material for: Comparative Study on Foaming Properties of Egg White with Yolk Fractions and Their Hydrolysates
Source: Foods. 2021 Sep 21;10(9):2238. doi: 10.3390/foods10092238 (PMC8468132; doi:10.3390/foods10092238)
Supplement: Supplementary file 1 [file foods-10-02238-s001.zip › foods-1375801-supplementary.pdf]

foods

Article

Comparative Study on Foaming Properties of Egg White with Yolk Fractions and Their Hydrolysates

Xin Li [1,*], Yue-Meng Wang [2], Cheng-Feng Sun [1], Jian-Hao Lv [1] and Yan-Jun Yang [3]

1 School of Life Sciences, Yantai University, Yantai 264005, China; cfsun@ytu.edu.cn (C.-F.S.); ljh17853509809@163.com (J.-H.L.)
2 School of Food and Biological Engineering, Yantai Institute of Technology, Yantai 264003, China; wangym0523@163.com
3 School of Food Science, Jiangnan University, Wuxi 214122, China; yanjunyangx@126.com
* Correspondence: nallyxin@163.com

Citation: Li, X.; Wang, Y.-M.; Sun, C.-F.; Lv, J.-H.; Yang, Y.-J. Comparative Study on Foaming Properties of Egg White with Yolk Fractions and Their Hydrolysates. *Foods* **2021**, *10*, 2238. https://doi.org/10.3390/foods10092238

Academic Editor: Susana Casal

Received: 26 August 2021
Accepted: 17 September 2021
Published: 21 September 2021

Publisher's Note: MDPI stays neutral with regard to jurisdictional claims in published maps and institutional affiliations.

Copyright: © 2021 by the authors. Licensee MDPI, Basel, Switzerland. This article is an open access article distributed under the terms and conditions of the Creative Commons Attribution (CC BY) license (http://creativecommons.org/licenses/by/4.0/).

Supplementary Materials:

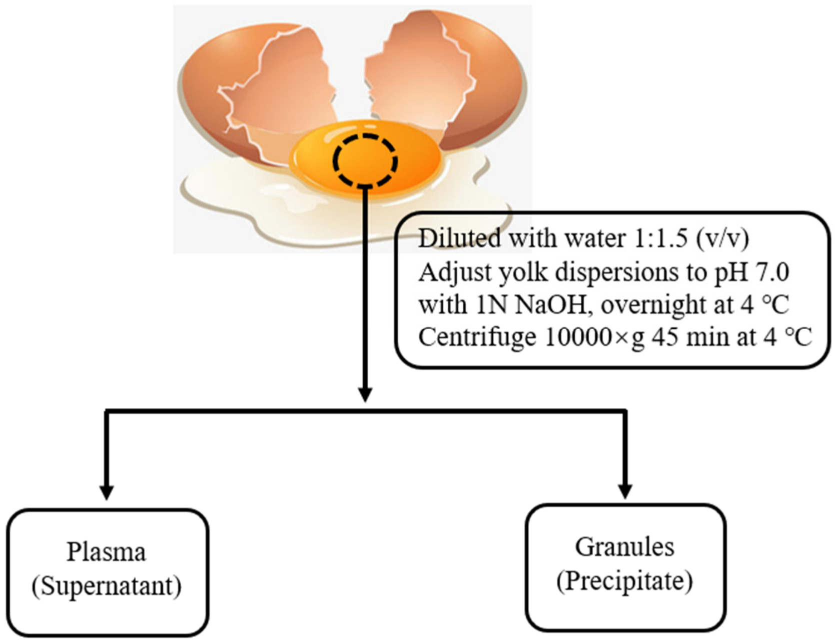

Figure S1. The preparation of egg yolk fractions (plasma and granules).
